# Characterization of the Blood Microbiome and Comparison with the Fecal Microbiome in Healthy Dogs and Dogs with Gastrointestinal Disease

**DOI:** 10.3390/vetsci10040277

**Published:** 2023-04-05

**Authors:** Elisa Scarsella, Giorgia Meineri, Misa Sandri, Holly H. Ganz, Bruno Stefanon

**Affiliations:** 1 AnimalBiome, 400 29th Street, Suite 101, Oakland, CA 94609, USA; 2 Department of Agricultural, Food, Environmental and Animal Sciences, University of Udine, Via delle Scienze 206, 33100 Udine, Italy; 3 Department of Veterinary Science, University of Turin, Largo Paolo Braccini 2, 10095 Grugliasco, Italy

**Keywords:** microbiome, blood, gut, gastroenteropathy, dogs

## Abstract

**Simple Summary:**

Blood has been considered a sterile environment. However, recent studies demonstrated the presence of bacterial DNA in healthy individuals. Most of the research has focused on human health, although this is an expanding topic in animal health. More needs to be done to understand the origin of bacteria found in blood, especially in relation to the host’s health status. This study aims to characterize the blood microbiome in healthy dogs and dogs with gastrointestinal disease and to compare the microbial blood population with the fecal microbiome. Results showed a clear separation of blood and fecal microbiome between the healthy and unhealthy groups, suggesting a translocation of the bacteria from the gut to the blood. More investigations are necessary to understand the bacteria’s origin and viability in blood.

**Abstract:**

Recent studies have found bacterial DNA in the blood of healthy individuals. To date, most studies on the blood microbiome have focused on human health, but this topic is an expanding research area in animal health as well. This study aims to characterize the blood microbiome of both healthy dogs and those with chronic gastro-enteropathies. For this study, blood and fecal samples were collected from 18 healthy and 19 sick subjects, DNA was extracted through commercial kits, and the V3-V4 regions of the 16S rRNA gene were sequenced on the Illumina platform. The sequences were analyzed for taxonomic annotation and statistical analysis. Alpha and beta diversities of fecal microbiome were significantly different between the two groups of dogs. Principal coordinates analysis revealed that healthy and sick subjects were significantly clustered for both blood and fecal microbiome samples. Moreover, bacterial translocation from the gut to the bloodstream has been suggested because of found shared taxa. Further studies are needed to determine the origin of the blood microbiome and the bacteria viability. The characterization of a blood core microbiome in healthy dogs has potential for use as a diagnostic tool to monitor for the development of gastro-intestinal disease.

## 1. Introduction

The gut microbiome is composed of bacteria, archaea, viruses, and eukaryotic organisms that reside in the gastrointestinal tract, and many interact with the host in a symbiotic fashion. For example, bacteria present in the gut are able to produce short-chain fatty acids (SCFA) that nourish the intestinal epithelium, while the epithelium produces mucus that feeds beneficial bacteria [1]. A growing number of studies have investigated the composition and the variation of the gastrointestinal microbial population in relation to healthy conditions and environmental factors for companion animals and livestock. The gut microbiome is responsible for meaningful crucial functions, the contribution of metabolic activities, protection against pathogens, signaling to the immune system, and direct or indirect effects of most physiological roles. Individual variation in microbiome composition is associated with factors, such as diet, age, geographic location, antibiotic use, and gender. The onset of innovative technologies has enabled an everyday use of molecular methods for non-culturable bacteria identification within the canine GI tract. The total microbial load is estimated to be negligibly higher than the number of host cells in the body (if the total host cell count included red blood cells) [2].

Recent studies suggested that blood uptakes healthy bacteria and their metabolites while circulating throughout the human body. Microorganisms in circulatory fluids of confined compartments (classically considered sterile), including spinal fluid and blood, are moderately new and scarcely studied concepts. [3,4,5,6]. In the past, the presence of bacteria in the bloodstream has always been considered an indication of infection and therefore of ongoing pathology. With the advancement of sequencing technologies, however, various studies in humans have shown that the presence of microorganisms in the blood is not always due to an infection, but to natural colonization of the bloodstream. The first evidence of the presence of microorganisms in healthy humans was reported in 1969 in a work by Tedeschi et al. [7], where the presence of a metaphorically active form of mycoplasma or bacterial L-form was detected. Subsequently, Domingue and Schlegel [8] found bacterial traces in the blood of healthy subjects. In 2001, Nikkari and colleagues [9] confirmed the presence of bacterial DNA in the blood of healthy subjects through qPCRs with specific primers. Using other specific primers, Moriyama et al., [10] also found different bacterial populations. Therefore, even if proof of the presence of microorganisms in blood would require visual and viability analyses, other studies have been conducted over the years that have demonstrated the presence of genetic material of microorganisms in the blood of healthy human subjects [11,12,13,14]. Over the years, the blood microbiome has also been observed and reported in various domestic animals and birds [15,16,17,18,19].

The origin of the microorganisms present in blood has yet to be ascertained. Mainly, it is hypothesized that the microorganisms spread through a translocation of the microbiome from different biological locations of the host organism that are richer in terms of bacterial presence or that enter during clinical procedures or from the environment through different types of injuries. For example, it is hypothesized that one of the main sources of enrichment of the blood microbiome is the oral microbiome. The bacteria that make up the oral population can enter the circulation when the tight junctions of the mucosal epithelial cells are compromised [20] or the gum is damaged. In addition to this, the skin microbiome can also enter the bloodstream when the epithelial barrier is damaged. A further hypothesis is the bacterial translation from the intestine to the blood, which would confirm the phenomenon of the “leaky gut”, or the translocation of bacteria from the intestine to the bloodstream due to a compromised epithelial barrier. However, the entry of microorganisms into the blood can also occur through various intestinal and circulatory cells [21]. An example is given by dendritic cells, which can carry microbial products through epithelial cells without affecting the tight junctions function [22]. In addition to this, the intestinal mucus-secreting goblet cells and the mucosal lymph cells that lay on Peyer’s patches can furthermore serve as microbial carriers from the intestine to the bloodstream [23].

Many studies have shown that the gut microbiome of healthy subjects is characterized by a high variability [24,25] and that it can represent a fingerprint that is able to distinguish each individual [26]. Several factors influence the microbial composition of the gastrointestinal tract, including diet, sex, age, and health status [27,28]. A strong similarity between humans and dogs has been ascertained over the years, and this suggests that the same factors that influence the human gut microbiome can also affect the dog’s gut microbiome [29].

A chronic enteropathy is characterized by persistent signs of gastrointestinal distress (such as vomiting and diarrhea) and can be categorized as food-, antibiotic-, and steroid-responsive, depending on the response to the treatment [30]. Idiopathic inflammatory bowel disease (IBD) is a subset of chronic enteropathies showing signs of inflammation of the gastrointestinal tract, symptoms with no apparent cause, and poor response to treatment [31]. The onset of IBD is still undefined, but there are several hypotheses that link this disease to intestinal dysbiosis, an immune response and genetic factors predisposing the development of this disease [32].

Numerous efforts have been made to establish a connection between the intestinal microbiome and the state of health of the host, but even today, there are still a limited number of studies that explore dysbiosis events concerning the blood microbiome only in the human area. The concept of dysbiosis refers to a change in the composition of symbiotic or commensal microbial communities in favor of the prevalence of a few pathogenic microorganisms [33]. This study aims to characterize the blood microbiomes of healthy dogs and dogs with chronic gastroenteropathies and to compare them with the fecal microbiome of the same subjects. To the best of the authors’ knowledge, this is the first attempt to characterize the blood microbiome in sick dogs. The purpose of this research is to provide further clarity on the origin of the blood microbiome, as well as being able to characterize this microbial population in depth so that it can be used as a diagnostic tool in the future.

## 2. Materials and Methods

### 2.1. Animals and Housing

A total of 36 dogs were enrolled from 4 veterinary clinics in 1 month. Both females (10 whole and 12 spayed) and males (10 whole and 3 neutered) were present in this study. Dogs were of different breeds and sizes and in the adult phase. The description of the population is in Supplementary Table S1. Dogs were divided on the basis of their health status at the moment of the visit. One group was categorized as “Healthy” (17 dogs) based on no clinical sign of sufferance at the moment of the visit, together with no antibiotic treatment in the previous 3 months, and no parasitic infection. Blood analysis was performed at the clinics in order to exclude other types of diseases, with each clinic’s own system that could differ from vet to vet. Blood analysis and others clinical reports were available for consult but not for publication due to restraint of privacy. The other group was categorized as “Sick” (19 dogs), since at the moment of the visit, the dogs showed clinical signs of sufferance, and the owners reported their symptoms on several occasions. All dogs that were part of the “Sick” group were from a clinic in Turin. Dogs were thus diagnosticated with a food responsive chronic gastrointestinal disease. The diagnosis was performed at the clinic by the clinic’s own type of analysis, and the dogs were not treated at the moment of the sampling collection. “Sick” dogs were put under dietary modification after the visit. Blood analysis was performed at the diagnosing clinic in order to exclude other types of diseases. Dogs were brought to the clinics for normal routine visits, where the veterinarian collected blood samples and asked the owners to collect feces for the study. Feces were stored at −20 °C until the time of the veterinarian visit. Whole blood was collected in a K3-EDTA tube with venipuncture from the radial vein after shaving the coat and carefully disinfecting the skin with chlorhexidine alcohol solution. The samples were promptly stored at −20 °C until analysis. Blood and feces were collected the same day to minimize the variability between these two types of biological matrices. Owners gave an informal consent, and no change in the environment and lifestyle was applied to the recruited dogs. All protocols and procedures for the animals complied with the Italian legislation on animal care and were approved by the ethical committee of the University of Udine (OPBA, Prot. N. 7/2019, issued on 28 June 2019). Both feces and blood were delivered to the laboratory within 15 days and processed within 3 days; there was no difference in the storage of the samples between healthy and sick dogs.

### 2.2. Fecal and Blood Microbial DNA Extraction and Sequencing

Microbial DNA was extracted by following the manufacturer’s instructions of two commercial kits, as explained in Scarsella et al. [18]. Briefly, prokaryotic DNA from fecal samples was extracted from approximately 150 mg of starting material, using the Quick-DNA Fecal/Soil Microbe Miniprep kit (Zymo Research; Irvine, CA, USA). DNA from blood samples was extracted from 200 μL of starting material, using an Exgene^TM^ Clinic SV kit (GenAll Biotechnology, Seoul, Republic of Korea). Both negative and positive controls were added to the analysis from the DNA extraction to the whole pipeline. Negative control was represented by molecular biology grade water, while positive control was a mock community standard of bacteria (Zymo Research; Irvine, CA, USA). DNA concentration was determined with a QubitTM 3 Fluorometer (Thermo Scientific; Waltham, MA, USA), and the V3-V4 regions of the 16S rRNA gene were amplified for library preparation, using a Nextera DNA Library Prep kit (Illumina; San Diego, CA, USA), following the manufacturer’s instructions and primers [34]. Sequencing of the amplicons was performed by NovaSeq 6000 (Illumina; San Diego, CA, USA) in 2 × 300 paired-end mode. The Quantitative Insights into Microbial Ecology (QIIME 2) [35] was used to process the raw sequences. After demultiplexing, sequenced reads that passed the quality check (Phred score  30) were annotated for 16S rRNA against the Silva database (version 138), with 99% identifying with reference sequences [36,37]. Detection and filtering of the chimeras were performed, together with the clustering of the remaining sequences into exact sequence variants by using an open-reference approach in QIIME 2.

### 2.3. Computational and Statistical Analysis

Alpha and beta diversity of the samples was analyzed using QIIME2 through the q2-diversity plug-in. Shannon and Evenness indexes were analyzed through a non-parametric Kruskall–Wallis test. A *p*-value below 0.05 was considered statistically significant, and one below 0.1 was considered a trend. Beta diversity was assessed using the Unweighted and Weighted UniFrac [38] and visualized by using the Principal Coordinate Analysis (PCoA) plot. Next, sample composition was analyzed in the context of categorical metadata using PERMANOVA [39]. The sequences for each sample were normalized to per mille abundance profiles, called relative abundances (RAs). Bacteria with RA lower than 1 per mille in 50% of the samples were excluded from the statistical analysis [17]. Venn diagrams were used for a clearer visualization of the shared taxa between feces and blood at the genus level, in healthy and sick dogs. The pathway abundances output was analyzed through Statistical Analysis of Metagenomic Profiles (STAMP), a software package for analyzing metagenomic profiles [40].

## 3. Results

### 3.1. Translocation of Bacteria from the Gastrointestinal Tract to the Blood

**Figure 1:**
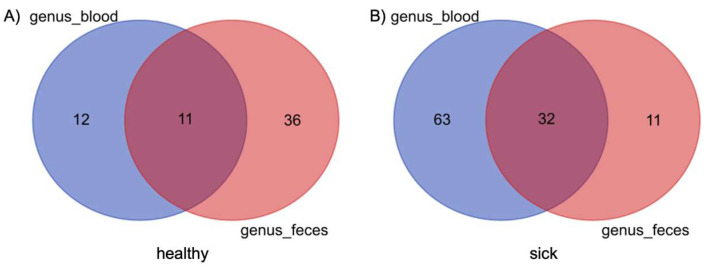
Composition similarity in the bacterial community at the genus level in blood and feces of healthy (**A**) and sick (**B**) dogs. Core genera are defined as genera that are found in both groups of dogs.

Figure 1 shows two Venn diagrams which compare the genera shared between blood microbiome and fecal microbiome in dogs with gastrointestinal diseases on the left and in healthy dogs on the right. The most evident result is that in the blood of sick dogs there is a higher bacterial diversity than in the fecal microbiome. For this type of analysis, the genera were filtered on the basis of their presence in a threshold of more than 50% of subjects. Therefore, it was possible to observe the presence of 95 different genera in the blood of more than 50% of sick subjects versus only 43 genera in more than 50% in the feces of sick dogs. These two matrices share 32 genera. The same type of analysis was conducted in healthy subjects, in which an opposite trend was instead observed. Only 23 genera were present in more than 50% of the blood of dogs, compared to 47 genera present in the feces of healthy dogs, taking into account the same percentage of presence. In this case, only 11 genera shared between the 2 matrices was highlighted. The list of taxa between the different matrices and between the two groups of dogs is given in Supplementary Table S2.

Table 1 shows the comparison between phyla detected in feces and blood. Bacteroidota and Fusobacteriota showed a statistically significant difference where there was a decrease in relative abundances in the feces of sick dogs and a significant increase (*p*-value less than 0.05) in the blood of sick dogs. Actinobacteriota exhibited a significant decrease of the relative abundance in the feces of sick dogs, but no fluctuations were observed in the blood for the two groups. Firmicutes and Proteobacteria did not show changes in the relative abundance of phyla in the feces of the two groups of dogs. Instead, a significant increase was observed for the relative abundances in the blood of the sick dogs.

The negative controls did not amplify, and the results regarding both negative and the mock bacterial community, used as an internal positive standard to validate the methodology and the reagents, confirmed the lack of contamination.

### 3.2. Characterization of the Blood Microbiome Related to the Gastrointestinal Health Status

**Figure 2:**
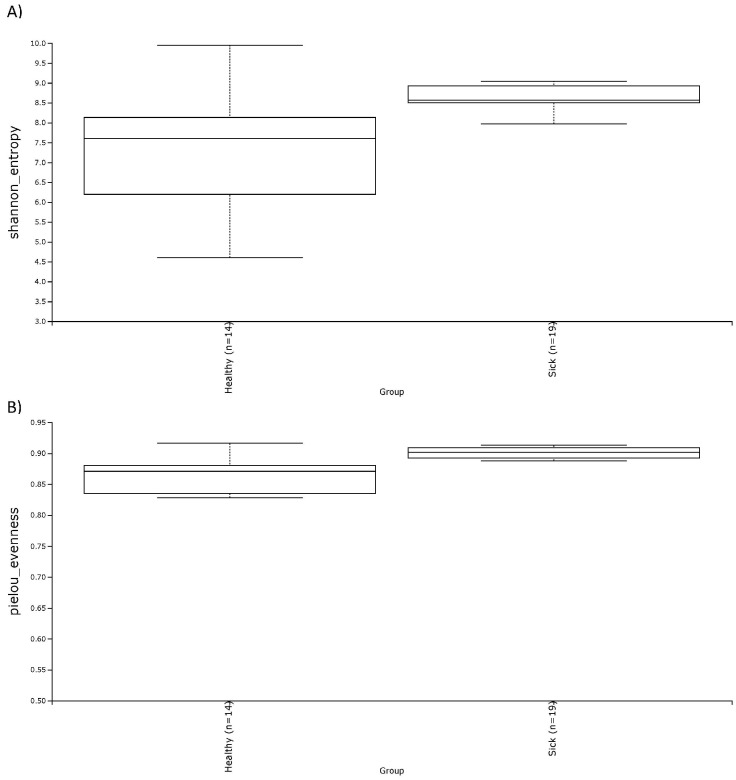
Alpha diversity, shown as the median of the Shannon index (**A**) and the Evenness index (**B**), in blood samples of healthy and sick dogs.

**Figure 3:**
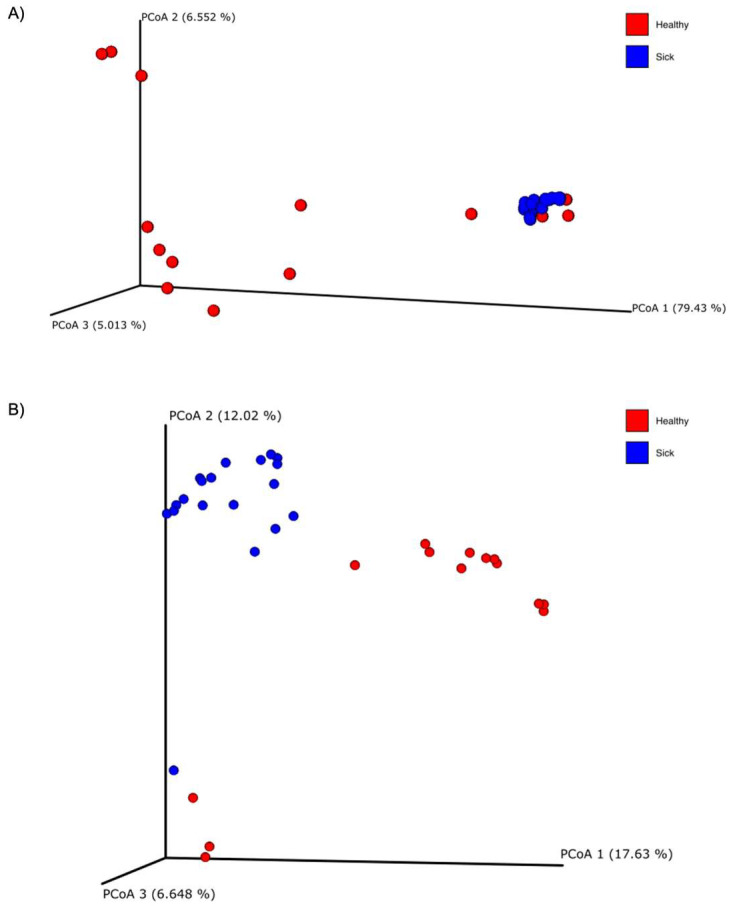
Principal coordinate analysis (PCoA) plots of the (**A**) weighted and (**B**) unweighted UniFrac beta diversity of the blood microbiome of healthy (red dots) and sick (blue dots) dogs.

Alpha diversity was calculated with the Shannon and the Evenness index in the blood microbiome of healthy and sick dogs. As shown in Figure 2, it is possible to observe significant differences in the blood microbial population for both indexes for healthy animals and animals with gastrointestinal disease. The alpha diversity was generally higher in sick dogs. This was confirmed by a non-parametric Kruskal–Wallis statistical analysis that showed a *p*-value below 0.05 in both the Shannon and the Evenness index. The results of the Kruskal–Wallis analysis are shown in Table 2. Moreover, the beta diversity highlighted a very distinctive clusterization of the subjects based on their gastrointestinal status. Both weighted and unweighted UniFrac dissimilarity matrix reported a separation of the dogs (Figure 3). The sum of the first two coordinates in the weighted UniFrac was 85.98%, whilst for the unweighted UniFrac it was 29.65%. In both cases, the dissimilarity between the two groups was explained with the first two eigenvectors. PERMANOVA analysis confirmed what was highlighted from the two PCoA plots, with a *p*-value below 0.05 (Table 2).

Table 3 showed the genera with significant changes in the blood of healthy dogs versus that of sick dogs. Among the genera of interest we found that *Acinetobacter*, *Escherichia*-*Shigella,* and *Turicibacter* showed a significant decrease in relative abundances in the blood of sick dogs. *Bifidobacterium*, *Megamonas*, *Prevotellaceae* group, *Sphingomonas,* and *Eubacterium coprostanoligenes* group showed a significant increase in the blood of sick dogs.

### 3.3. Characterization of the Fecal Microbiome Related to the Gastrointestinal Health Status

**Figure 4:**
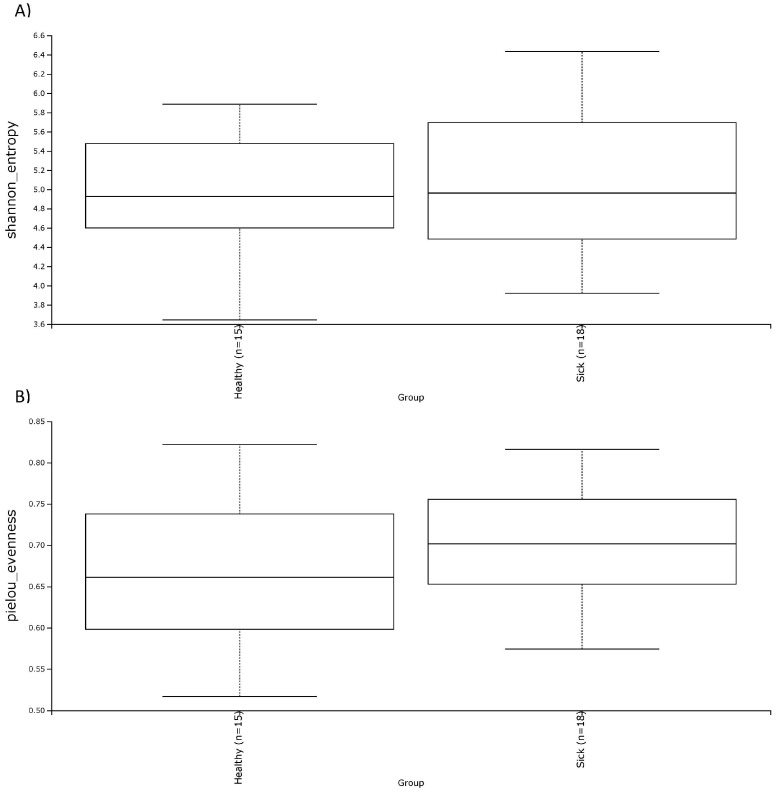
Alpha diversity, shown as the median of the Shannon index (**A**) and the Evenness index (**B**) in fecal samples of healthy and sick dogs.

**Figure 5:**
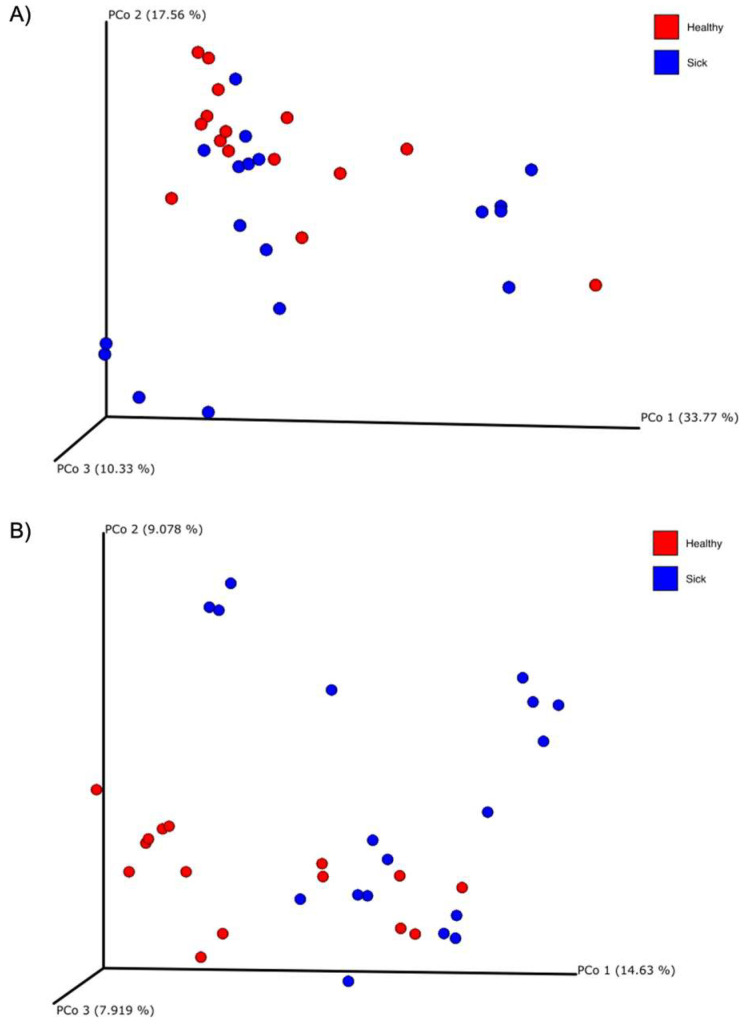
Principal coordinate analysis (PCoA) plots of the (**A**) weighted and (**B**) unweighted UniFrac beta diversity of the fecal microbiome of healthy (red dots) and sick (blue dots) dogs.

In terms of alpha diversity, both the Shannon and the Evenness index did not show noticeable differences for the two groups of dogs (Figure 4). This was also confirmed by the non-parametric test of Kruskal–Wallis (Table 4) which showed a *p*-value above 0.05. Regarding beta diversity, there was a trend of diversification of the subjects, especially in the unweighted UniFrac dissimilarity matrix (Figure 5). In fact, this trend is confirmed by PERMANOVA analysis, which highlighted a significance both in the weighted UniFrac dissimilarity matrix (*p*-value = 0.039) and especially in the unweighted UniFrac dissimilarity matrix (*p*-value = 0.001). These results are reported in Table 2.

Table 5 shows the fecal genera showing significant differences between the two groups. *Bacteroides*, *Collinsella*, *Fusobacterium*, *Slackia*, *Sutterella*, *Ruminococcus gnavus* group, and *Ruminococcus torques* group show a decrease in relative abundances in the feces of sick dogs. *Megamonas* is the only genus that shows a significant increase in relative abundances in the feces of sick dogs.

### 3.4. Characterization of Predicted Functionality on Gut and Blood Microbiome in Healthy and Sick Dogs

**Figure 6:**
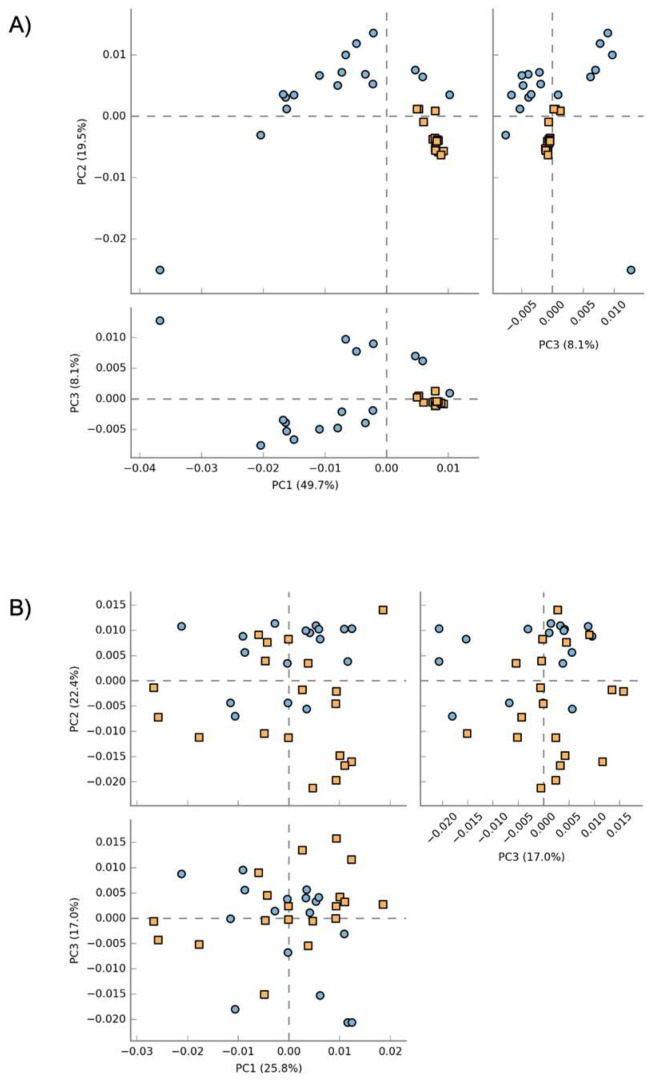
Principal coordinate analysis (PCA) plot of the functional pathways predicted by Picrust2 of the blood microbiome (**A**) and the fecal microbiome (**B**). Blue dots represent healthy dogs, while orange squares represent sick dogs.

Principal component analysis (PCA) plots of the predicted pathway abundances regarding the blood and the fecal microbiome of healthy and sick dogs are shown in Figure 6. A clustering of the healthy and sick dogs was clearly observed when the functional pathways of the blood microbiome were analyzed. The variability was explained with a high percentage with the first two components for a total of 69.2% (PC1 = 49.7%; PC2 = 19.5%). After performing ANOVA on the functional pathways related to the blood microbiome, and after Bonferroni correction, the remaining active features were 88. The same did not happen for the fecal microbiome, where the healthy and sick dogs did not clearly cluster apart. The variability was explained with lower percentage in the first three components (PC1 = 25.8%; PC2 = 22.4%; PC3 = 17%). ANOVA analysis was also performed for these pathway abundances, and after Bonferroni correction, 0 features remained.

## 4. Discussion

To the best of our knowledge, this is the first study comparing the blood microbiome of healthy dogs and dogs with gastrointestinal disease. The Venn diagrams, shown in Figure 1, denoted that there are a certain number of shared genera between feces and blood, 11 taxa for the healthy animals and 32 taxa for the sick dogs. This observation is also confirmed by previous studies on both healthy dogs and cattle [17,18]. What was interesting was that dogs with gastrointestinal disease showed a considerably higher number of shared genera between blood and feces, together with a decrease of the number of taxa that are part of the gut core microbiome, against an increase in those present in the blood. This result leads us to hypothesize that in the sick group there was what is called “leaky gut”, a condition widely studied in humans and dogs and which is oftentimes associated with IBD and other interconnected enteropathies [41,42,43,44]. Despite the presence of the shared taxa, it is hard to confirm the occurrence of microbiome translocation from one site to another. External factors, such as contamination of environmental bacteria, could have led to an artificial appearance of microbiome in the blood. Even if we cannot completely exclude this hypothesis, we were confident that this was not the case since we ran a negative control from the DNA extraction to the sequencing. Moreover, healthy dogs were screened with blood tests and recruited based on no visible diseases or syndromes at the time of the visit, although we cannot completely exclude the presence of pathologies that could have influenced the microbiome composition, and this constitutes a limitation for this study.

In this study, a significantly higher alpha diversity of the microbial blood population was observed in healthy dogs than in sick ones. Together with the analysis of the shared genera, this result confirmed the occurrence of bacterial transfer from the gut to the blood. Regarding the alpha diversity of the gut microbiome of the two groups, there were no significant differences between healthy and sick dogs for both the Shannon and the Evenness indexes. Results that are consistent with our outcomes come from a study on the fecal microbiome of healthy dogs with acute diarrhea and IBD, where the OTU index, the Shannon index, and the Chao1 index were not significant among the various groups considered [45]. Other studies, on the other hand, reported significant differences between the various alpha diversity indices analyzed. For example, Vázquez-Baeza et al. [46] found significantly lower alpha diversity by using the Faith’s phylogenetic index in dogs with IBD compared to healthy dogs. Isaiah et al. [47] reported a significantly lower Shannon index, Chao1 index, and observed OTU index in treated and untreated dogs with pancreatitis compared to the healthy dog group. In another study, where three different factors—breed, dietary protein, and neutering status—were analyzed as potential candidates to influence the gut microbiome in both healthy and IBD dogs, the authors observed a decline in alpha diversity using the Shannon index in dogs with IBD [48].

Through the principal coordinate analysis (PCoA) plot, we are able to notice a clustering of sick dogs against healthy dogs, both regarding the microbiome of the blood (Figure 3) and that of the feces (Figure 5). The blood microbiome in the two groups is made up of an extremely different bacterial population. This leads us to think that there is a level of normality; that is, that some taxa are naturally present in the blood of healthy subjects. Historically, blood has been considered a sterile environment; with time, the nonpathogenic condition of the microbial population presence in the blood was defined as the “dormant phase” of bacteria [49]. However, some of these taxa could start to reproduce and become active again. In this way, some of these bacteria can be transported to the peripheral tissues and organs, inducing chronic disease [50]. Few studies have been conducted on animals. In the first research regarding dogs, the presence of bacterial DNA in the bloodstream of healthy subjects was confirmed [17]. From this study, it was speculated that the origin of the blood bacterial DNA was the gut microbiome, given that a shift in bacterial composition based on the dog’s diet was observed. A recent study on goats confirmed the presence of bacterial DNA in the blood of both healthy goats and goats infected with *H. contortus*, a parasite that infects the gastrointestinal tract. The authors also confirmed the difference in the microbial composition of the two groups under examination, as well as the goats infected with *H. contortus* and treated with different treatments [19]. Another research study considered the fecal, blood, and milk microbiome in healthy cows, cows with mastitis and cows at risk of mastitis [18]. Although no significant difference was observed in terms of beta diversity in the blood microbiome in the three tested groups, it was still possible to confirm the presence of bacterial DNA in the blood in both healthy and cows with mastitis.

Although the alpha diversity of the fecal microbiome was not significant for the two groups, this was not true for the beta diversity. Figure 5 shows the PCoA plots about the weighted and unweighted UniFrac matrix of the gut microbiome. This observation confirmed what was already seen in many other studies conducted in dogs. In research from Suchodolski et al. [45], where healthy dogs were compared with dogs divided for different enteropathies, through the weighted UniFrac matrix, a statistically significant separation of healthy dogs from dogs with acute hemorrhagic diarrhea (AHD) was observed and at the same time, of healthy dogs from dogs with acute non-hemorrhagic diarrhea (NHD). In the end, it was observed between dogs with AHD and dogs with NHD. However, this significance was not observed when comparing healthy dogs with dogs with IBD. Vázquez-Baeza et al. [46] instead observed a statistically significant clustering of the group of healthy subjects compared to dogs with IBD, through the unweighted UniFrac distance analyzed with the PERMANOVA analysis. A study in dogs with pancreatitis revealed that there was a statistically significant difference in the gut microbiome between healthy dogs and dogs with treated and untreated pancreatitis in terms of beta diversity calculated with the UniFrac unweighted matrix [47]. Calalang et al. [48] observed a separation between healthy dogs and dogs with IBD through the UniFrac weighted matrix, but this observation was not confirmed by any statistical analysis. Lastly, a recent work on FMT recipient dogs showed that healthy dogs and dogs with chronic enteropathies have a different microbial composition through four different indices of beta diversity, including the weighted and unweighted UniFrac distances [51]. This latest study confirms exactly what has been currently observed with this research. Although some of these studies seemed to confirm what was observed in this current case, this study suffered from some limitations; the dogs were diagnosticated by each clinic based on their own method, and we cannot completely exclude the co-occurrence of other pathologies.

Actinobacteria showed a significant decrease in the fecal microbiome but not in the blood microbiome. It was shown in a human study that the oxygen gradient closer to the GI mucosa selects for aerotolerant species, such as Proteobacteria and Actinobacteria, that express higher levels of oxygen-detoxifying catalases than other lumen residents [52]. Usually, microorganisms are closer to the epithelium in inflamed guts [53], and the colonization of the gut from some ectopic pathogens requires the presence of gut inflammation [54]. Modification in the inflammation-associated mucosal microbiota may increase because mucus is physically weaker in this situation [55]; thus, pathogens could have easier access to the host epithelium. Closer proximity and access can enhance damage to the host, including the exacerbation of bacterially induced inflammation [56]. Firmicutes and Proteobacteria do not show any significant variation between the two groups in the fecal microbiome, but instead they are significantly increased in the blood of sick animals. Bacteroidota and Fusobacteria showed an opposite trend, where it was possible to observe a significant decrease in terms of RAs in the feces of the sick dogs. Meanwhile, a significant increase was noted in the blood of the same group of dogs. All of these observations could led to a translocation of bacteria from the gut to the blood of dogs with gastroenteropathies, although it is not possible to completely confirm that. Moreover, it is also possible to conclude that the presence of bacteria in blood is common even in healthy subjects and that there is a threshold for normality in the abundances of these taxa. The term dysbiosis refers to a change of the normal composition of the gut microbiome into a microbial imbalance in the digestive tract. This change of composition often led to a pathology. In this case, it is possible to refer to the term “atopobiosis” that means a translocation of the microorganisms from their original location to another one [57]. In the case of chronic enteropathies, it appears that the gut microbiome could be the main source of the blood microbiome. There are three possible points of entrance for the microorganisms into blood [21]. The first way is by dendritic cells via processes between epithelial cells. The second is by injured or inflamed epithelium with a consequently dysfunctional epithelial barrier. The third and the last one is though M cells overlying Peyer’s patches as specialized cells providing access of microbial products to antigen presenting cells.

There is still much to be investigated in order to understand the origin of the bacterial presence in blood, both in healthy and sick dogs. First of all, it is necessary to understand the role that these microorganisms play in healthy subjects and evaluate the normal threshold of the bacterial presence and their viability. Second, we need to find out the role they play in sick subjects. Understanding the composition of the blood microbiome in individuals with gastroenterological diseases is the first step, after which it must be put in the context of other diseases in order to use the blood microbiome as a fast and reliable diagnostic tool. Further research needs to be conducted, but the first steps to understand the composition and origin of the blood microbiome, as well as its role in the course of disease, have been made in humans, domestic animals, and now companion animals as well.

## Figures and Tables

**Table 1:** Kruskal–Wallis non-parametric test of the relative abundances (RAs) of the phyla characterizing the fecal and the blood microbiome of healthy and sick dogs. Mean and standard deviation (SD) are reported. *p*-value below 0.05 denotes significant differences of the means.

		Healthy		Sick		*p*-Value
		Median	SE	Median	SE	
Actinobacteriota	Feces	46.1	8.1	3.4	4.6	0.001
	Blood	14.3	8.1	9.4	7.9	0.579
Bacteroidota	Feces	38.1	13.6	6.4	2.6	0.005
	Blood	2.4	16.2	167.2	7.0	<0.0001
Firmicutes	Feces	749.0	31.1	705.7	48.6	0.788
	Blood	33.5	32.8	382.1	8.5	<0.0001
Fusobacteriota	Feces	42.0	10.8	4.8	5.1	0.038
	Blood	0.0	0.2	2.9	0.7	0.000
Proteobacteria	Feces	4.7	9.0	1.1	4.5	0.145
	Blood	23.6	28.3	197.4	3.8	<0.0001
Cyanobacteria	Feces	n.a.	n.a.	n.a.	n.a.	
	Blood	0.0	0.5	0.7	0.2	0.103

**Table 2:** Non parametric Kruskal–Wallis test of the Shannon and Evenness alpha diversity indexes and PERMANOVA analysis of the weighted and unweighted UniFrac dissimilarity matrix on the blood microbiome of healthy and sick dogs. *p*-value below 0.05 denoted significance.

	Healthy (Median)	SE	Sick (Median)	SE	H	*p*-Value	*q*-Value	Pseudo-F
Shannon index	7.60	0.52	8.56	0.14	4.78	0.029	0.029	
Evenness index	0.87	0.03	0.90	0.004	10.99	0.0009	0.0009	
PERMANOVA weighted UniFrac						0.001	0.001	30.56
PERMANOVA unweighted UniFrac						0.001	0.001	4.93

**Table 3:** Kruskal–Wallis non-parametric test of the relative abundances (RAs) of genera, characterizing the blood samples of healthy and sick dogs. Mean and standard deviation (SD) are reported. *p*-value below 0.05 denotes significance.

	Healthy		Sick		*p*-Value
	Median	SE	Median	SE	
Acinetobacter	7.7	4.1	0.0	0.1	<0.0001
Alistipes	0.0	0.4	23.9	1.8	<0.0001
Alloprevotella	0.0	0.4	9.8	1.0	<0.0001
Bacteroidales_RF16_group	0.0	0.0	11.9	0.7	<0.0001
Bacteroides	0.0	16.1	26.8	1.0	0.001
Bifidobacterium	0.3	0.6	3.1	7.0	<0.0001
Blautia	0.0	1.8	4.6	1.8	0.010
Christensenellaceae_R-7_group	0.0	0.5	8.1	0.9	<0.0001
Clostridia_UCG-014	0.0	0.4	12.7	1.1	<0.0001
Collinsella	0.0	7.1	6.5	1.8	0.011
Cutibacterium	6.2	5.7	0.0	0.2	0.000
Escherichia-Shigella	0.0	4.6	2.4	0.6	0.000
Family_XIII_AD3011_group	0.0	0.1	3.2	0.5	<0.0001
Fusobacterium	0.0	0.2	2.9	0.7	0.000
Izemoplasmatales	0.0	0.1	2.7	0.4	<0.0001
Megamonas	0.0	0.1	3.1	0.3	<0.0001
Monoglobus	0.0	0.0	8.7	0.6	<0.0001
Muribaculaceae	0.0	0.1	4.9	0.5	<0.0001
Prevotellaceae_UCG-001	0.0	0.0	2.2	0.3	<0.0001
Prevotellaceae_UCG-003	0.0	0.0	14.2	1.4	<0.0001
Rikenellaceae_RC9_gut_group	0.0	0.4	35.7	2.3	<0.0001
Ruminobacter	0.0	0.0	6.7	0.5	<0.0001
Ruminococcus	0.0	0.1	6.8	0.6	<0.0001
Sphingomonas	0.0	1.3	146.4	3.0	<0.0001
Turicibacter	0.0	0.9	1.4	0.2	0.014
UCG-005	0.0	0.5	56.5	3.1	<0.0001
UCG-010	0.0	0.0	84.6	6.1	<0.0001
[Eubacterium]_coprostanoligenes_group	0.0	0.1	21.5	1.4	<0.0001
p-2534-18B5_gut_group	0.0	0.1	2.3	0.3	<0.0001

**Table 4:** Non-parametric Kruskal–Wallis test of the Shannon and the Evenness alpha diversity indexes, and PERMANOVA analysis of the weighted and unweighted UniFrac dissimilarity matrix on the fecal microbiome of healthy and sick dogs. *p*-value below 0.05 denoted significance.

	Healthy (Median)	SE	Sick (Median)	SE	H	*p*-Value	*q*-Value	Pseudo-F
Shannon index	4.93	0.18	4.96	0.18	0.064	0.800	0.800	
Evenness index	0.66	0.02	0.70	0.02	1.099	0.294	0.294	
PERMANOVA weighted UniFrac						0.026	0.026	2.43
PERMANOVA unweighted UniFrac						0.001	0.001	2.63

**Table 5:** Kruskal–Wallis non-parametric test of the relative abundances (RAs) of genera, characterizing the fecal samples of healthy and sick dogs. Mean and standard deviation (SD) are reported. *p*-value below 0.05 denotes significance.

	Healthy		Sick		*p*-Value
	Median	SE	Median	SE	
*Bacteroides*	17.9	9.9	2.9	1.2	0.001
*Collinsella*	33.4	8.4	0.0	4.0	0.000
*Fusobacterium*	42.0	10.8	4.8	5.1	0.038
*Megamonas*	5.0	3.5	0.1	34.3	0.002
*Slackia*	5.4	1.3	0.4	0.7	0.009
*Sutterella*	0.6	1.2	0.0	0.2	0.020
*[Ruminococcus]_gnavus_group*	16.0	7.0	4.9	3.0	0.011
*[Ruminococcus]_torques_group*	3.4	0.9	0.0	0.4	0.002

## Data Availability

Raw data supporting the conclusions of this article will be made available by the authors, without undue reservation.

## Supplementary Materials

The following supporting information can be downloaded at: https://www.mdpi.com/article/10.3390/vetsci10040277/s1, Table S1: Description of the dog population analyzed in the study. Table S2: List of genera shared and not between blood and fecal material.
